# Social Media, Stereotypes, and the Acknowledgement of War Crimes

**DOI:** 10.1080/17502977.2024.2316747

**Published:** 2024-03-20

**Authors:** Sanja Vico

**Affiliations:** Department of Communications, Drama and Film, University of Exeter, Exeter, United Kingdom

**Keywords:** Social media, stereotype threat, cultural intimacy, mediated visibility, acknowledgement, war

## Abstract

Human rights activists increasingly employ social media to promote post-conflict justice and reconciliation. This study asks what role social media play in facilitating the acknowledgement of war crimes committed by members of one’s ethnicity and what the implications of mediated visibility are. It finds that people are less willing to acknowledge ingroup responsibility for war crimes on social media because they fear being negatively stereotyped by foreign audiences and reputationally undermined. The study sheds light on the unintended negative consequences of mediated visibility of war crimes and counters presumptions of digital universalism showing that implications of visibility are context dependent.

## Introduction

Human rights activists have been increasingly employing social media to tackle the denial of war crimes by raising awareness of war crimes, mobilising public support, and bringing communities together in conversations (Fridman and Ristic 2020; Kasadha 2020). Denial of war crimes causes harm to the survivors, hinders reconciliation between the confronted ethnic groups, and increases the risk of new violence (Minow 2002, 15–16). The visibility that social media afford in these contexts has been discussed in positive terms. Negative or unintended consequences of mediated visibility on discouraging acknowledgement of ingroup responsibility have been overlooked. This study concerns the role of social media in facilitating the acknowledgement of war crimes committed by an ingroup and the implications mediated visibility has in this respect. Social media position people on a global stage vis-à-vis many different others (Miller and Slater 2000), the so-called ‘international society.’ The international society has been based on the stigmatisation of norm-violating states (Adler-Nissen 2014; Hatuel-Radoshitzky and Jamal 2022). Individuals may internalise this stigma when they perceive to be viewed in the light of their national identity (see Vico 2020a) and realise their fate may depend on that of their country of origin. As the Latin proverb has it, ‘Nomen est omen,’ meaning one’s name is one’s destiny. Consequently, individuals may strive to defend their country’s reputation on an international stage, which may have implications on how they discuss the legacy of war on social media as opposed to face-to-face interactions.

To explore the role of social media in facilitating the acknowledgement of ingroup responsibility for war crimes, I look at differences between intra-ethnic interactions on social media and those conducted face-to-face in Serbia concerning the #sedamhiljada initiative. This initiative was launched on Twitter (now known as X) in 2015 to pay tribute to the victims of the Srebrenica genocide, where a Bosnian Serb army killed over 8000 Bosniak men and boys in July 1995. It was an example of public acknowledgement of ingroup responsibility aimed at mobilising the public to engage in a public performance on the main square in the capital Belgrade. I conducted focus groups to study face-to-face interactions and collected data on Facebook and Twitter interactions through the search of keywords such as Srebrenica and #sedamhiljada. I then applied discourse analysis to interpret textual data from face-to-face and social media interactions.

I find there are differences between how people discuss the war legacy publicly – on social media, and privately – in face-to-face focus groups, which is in line with insights from other studies published in this volume – including parliamentarian questions in Croatia (Kostovicova and La Lova forthcoming) and community photography initiative in Rwanda (Fairey forthcoming). In face-to-face interactions, people were more willing to acknowledge ingroup responsibility for war crimes, while on social media they closed up. I tried to understand why this was happening. What I observed was that most participants feared that the acknowledgement could reinforce the perceived negative stereotype of their ethnic group among international audiences because of this group’s war conduct. This fear hindered the acknowledgement but to a different extent depending on whether the war legacy was discussed in face-to-face interactions with a smaller group of compatriots or on social media where they were exposed to wider audiences, including foreigners. Exposing national weaknesses to foreign audiences was perceived as reputationally damaging, not just for the nation, but also for an individual. These findings show how the social media affordance of visibility may have unintended implications of discouraging acknowledgement of war crimes. Mechanisms through which I explain the observed patterns are the theory of stereotype threat (Inzlicht and Schmader 2011) and the concept of cultural intimacy (Herzfeld 1996). In what follows, I first discuss these theories and concepts, followed by a discussion on the procedure and rationale of conducting focus groups and discourse analysis, and the analysis of the findings. The empirical section is organised around three dominant manifestations of the stereotype threat in a post-conflict context that I identified inductively both in social media and focus group data.

## Stereotype threat and identity

The first theory I draw on to explain why people were less willing to acknowledge war crimes in interactions on social media is that of stereotype threat when acts are public. While we know that conflicts fuel negative stereotypes of the groups involved and that this hinders reconciliation between the groups (Bar Tal and Labin 2001; Maoz 2000), what is less known are the implications of the perception of being negatively stereotyped because of ingroup war conduct on the acknowledgement of this conduct. Stereotyping is an automatic and subconscious act of categorising experiences and the world around that accentuates similarities between elements of one group and differences between groups of elements (Hogg and Abrams 2003, 339–340). This creates polarities often turned into facts that then become ‘a part of a moral universe’ (Herzfeld 1996, 165). In a world where nations are still the dominant form of belonging (Calhoun 2017; Ichijo 2017), people all the time ascribe certain meanings to one another depending on where they come from or which passports they have (Anderson 1991; Trandafoiu 2013). How one is perceived may have material and symbolic implications on one’s life (Trandafoiu 2013, 124). As Herzfeld highlights, ‘stereotyping is a discursive weapon of power’ (1996, 157). A concern that a negative image of one’s group upheld by an outgroup may affect one personally is called a ‘stereotype threat.’ A stereotype threat is a type of identity threat that arises when people sense they could be ‘devalued based on their group’ (Cohen, Purdie-Vaughns, and Garcia 2012, 282). This theory argues that if people think they can be judged based on negative stereotypes about their group identity rather than on individual merits, they will avoid activities that may trigger the stereotype (Inzlicht and Schmader 2011). It also gives rise to an ‘I am us’ mindset (Cohen and Garcia 2005, 567), even if unwittingly. As a result, individuals may feel compelled to defend their country’s international image.

This theory has been mainly applied to classroom settings to study the impact of racial and gender-based stereotypes on students’ performance. These studies find that a fear of being stereotyped undermines students’ performance and leads to avoidance and even withdrawal from the pursuit of a degree (Inzlicht and Schmader 2011). Applied in post-conflict contexts, people may avoid acknowledging ingroup responsibility for war crimes if they fear such an acknowledgment could reinforce a negative image of their group and in turn undermine their global social standing. Public acts, such as interactions on social media, can lead to two subtypes of stereotype threat – own-reputation threat and group-reputation threat (Shapiro 2011). An own-reputation threat arises when one fears they could be judged or treated negatively by an outgroup because of a negative stereotype of their group. A group-reputation threat refers to a fear that one’s performance may reinforce a negative stereotype about one’s group, ‘the fear of being a bad ambassador.’ Shapiro contends that identification with one’s group is not required in the instance of an own-reputation threat unlike a group-reputation threat (2011, 76). I argue that both types of stereotype threat can be experienced regardless of the strength of one’s national or ethnic identity, because in an international context, for many, individual reputation is hardly fully independent from that of their group and country (cf. Cohen and Garcia 2005), as already explained.

Most studies that strive to explain impediments to the acknowledgement of ingroup war crimes have focused on psychological implications and motivations, such as a threat to self-esteem, overlooking symbolic implications such as that to one’s reputation and social status. An example of the former is the self-affirmation theory, which postulates that people are more willing to accept criticism of one aspect of their identity if it does not undermine their overall self-esteem (Sherman and Cohen 2006). This theory has been applied in post-conflict contexts and studies show that individuals fear the acknowledgment may jeopardise their self-esteem, especially when their ethnic identity is central to them, but once they are prompted to detach their values from their ethnicity, they are more likely to acknowledge ingroup responsibility (Cehajic-Clancy et al. 2011). Similarly, other studies show when one nation is reminded of its positive attributes and accordingly their national self-esteem is boosted, hostility towards other nations decreases and it gives rise to positive sentiments (Chung and Woo 2015).

What we learn from these studies is that people seek affirmation, but they do not only long for affirmation in their own eyes – to boost their self-esteem, as these studies have shown, but also affirmation in the eyes of others – to boost their reputation and social standing. It can be argued that a failure to acknowledge war crimes can be motivated by a threat to social status on a global stage. As Rivera notes (2008), war crimes are reputation-damaging events, and countries’ economic development, such as their ability to attract foreign investments and tourists, largely depends on their reputation. People’s livelihoods may consequently be affected too (Rivera 2008, 628). Apart from these material implications, stereotype threat also has symbolic implications. People may be drawn by a desire to be accepted as equals in a society they feel they belong to – such as the international community of consolidated democracies or developed European nations (see Vico 2020b). In this sense, an audience of cultural outsiders as the evaluative other plays a key role in understanding differences between people’s willingness to acknowledge war-time atrocities publicly as opposed to doing so privately.

## Cultural intimacy and affordances

Another theory that helps us further explain the observed differences between how people address war crimes on social media as opposed to in face-to-face encounters is that of cultural intimacy. Cultural intimacy refers to differences between ‘official self-representation and what goes on in the privacy of collective introspection’ (Herzfeld 1996, 14). Maio, Haddock, and Verplanken (2019, 81) also observe this distinction between public and private behaviour and argue that private acts are led by attitudes whereas public acts are led by an awareness of one’s public image. In other words, people use their audiences as a guide to their behaviour in their public acts. Cultural intimacy, more specifically, is a ‘recognition of aspects of a cultural identity that are considered a source of external embarrassment but that nevertheless provide insiders with their assurance of common sociality’ (Herzfeld 1996, 3). Herzfeld writes that Greeks’ attempts to regulate their national image abroad are an example of this. Greeks, according to Herzfeld, have an expression for not discussing perceived national weaknesses before foreign audiences that says, ‘matters of the house should not be exposed in the public sphere,’ whereby a nation is likened to a family (103). It is this reliance on the language of kinship that creates a sense of loyalty to the nation which explains why people may feel compelled to defend a compromised national image (172).

Studies that have applied the concept of cultural intimacy in the context of social media make an argument about social media as spaces for bonding for otherwise marginalised transnational groups (Min, Jin, and Han 2019) and for national identity construction through self-mockery (Bergere 2020; Kania-Lundholm and Lindgren 2017; Yang, Tang, and Wang 2015) and hashtag alignments (Shenton 2020). However, the concept has been overstretched in this literature insofar as the distinction between intimacy and familiarity has been blurred. While familiarity refers to local knowledge, intimacy usually involves some level of secrecy before outsiders. Yang, Tang, and Wang (2015) argue that Chinese social media users use the word *diaosi* as self-mockery which provides them with a sense of national intimacy, but which is taken as an insult if directed by an outsider. Herzfeld (1996) argued that insiders could only criticise their group privately. The film Zorba the Greek by Michael Cacoyannis (a Greek person) encountered a storm of criticism domestically because it was considered ‘demeaning to the Greek image abroad’ (Herzfeld 1996, 97). The literature on international relations also finds evidence of incongruities between how nation-states present themselves internationally and how they understand themselves privately and argues that the audiences of outsiders – the international society – play a pivotal role in the self-presentation of nation-states (Rivera 2008; Subotic and Zarakol 2012). This concept then suggests that greater visibility and exposure to international audiences on social media may affect people’s willingness to acknowledge ingroup responsibility for war crimes.

Subotic and Zarakol (2012) find that Serbia intimately disagrees with the ruling of the International Criminal Tribunal for the former Yugoslavia (ICTY), while it publicly cooperates with the ICTY in terms of extraditing suspects for war crimes. However, the very opposite dynamic may also be plausible: individuals may intimately condemn misconduct and war-time atrocities of their nation or ethnic group, but may not be willing to do so publicly, such as on social media, because of the risk of a stereotype threat, as discussed. People seek recognition and respect at the international level. While visibility on social media can be positively attributed to raising awareness of alternative discourses about the war (Fridman and Ristic 2020), it also opens one to criticism and can increase the risk of a stereotype threat. Visibility does not always lead to empowerment, recognition, and positive change but, in certain socio-political contexts, can exacerbate prejudice and have negative effects (Mihelj et al. 2023). For instance, in countries where political elites promoted homophobia and where media freedoms were limited, mediated visibility of same-sex relationships was found to have amplified the prejudice (ibid.). Social media affordances such as visibility are not universal, they can have different meanings to different people, which makes affordances contextual or relational (Willems 2021). This perspective counters the presumptions of digital universalism – that technological affordances affect all people in all contexts in the same way (Chan 2014, 178). The limits of tackling war-time atrocities on social media may not strictly be tied to technical aspects of social media but can be more related to people’s practices on these media and dependent on existing socio-cultural dynamics and power relations in a particular context. In post-conflict contexts, greater visibility may be unwanted due to greater scrutiny by the international society, as a moral arbitrator that one’s international recognition and social standing depend on. Hence, this paper aims to investigate the implications of visibility on social media on how people discuss the war legacy on social media and the role of social media in facilitating the acknowledgement of ingroup war crimes, largely unexplored and little-understood questions to date. To this end, I will specifically consider intra-ethnic interactions in Serbia on social media and in face-to-face encounters.

## Research design and methodology^1^

Serbia is a typical case of a post-conflict country (see Levy 2008), insofar as many post-conflict societies, including other former Yugoslav republics, experience widespread denial of war crimes committed by their group (see Hermann 2004; Milanovic 2016). While ICTY ruled in 2004 that the crimes committed in Srebrenica in July 1995 by the Bosnian Serb army against over 8000 Bosniak men and boys constitute genocide, the Serbian parliament brought a declaration about the Srebrenica massacre in 2010, denying the established nature or intent of these crimes. The #sedamhiljada initiative was a prominent example of public acknowledgement of ingroup responsibility and outgroup suffering that aimed to mobilise the support of the Serbian public for acknowledgement. The tweet that sparked the initiative was a spontaneous reaction of a former Belgrade journalist to the performance of students at the University of Zagreb who laid down on a public square in solidarity with terrorist attack victims of Garissa University in Kenya. He wrote ‘Imagine 7000 of us lay down in front of the National Assembly of Serbia to mark the anniversary of Srebrenica,’ which led to the campaign hash-tagged seven thousand. Twitter and Facebook were the main social media platforms employed to publicly promote this campaign in 2015. Instagram was not predominantly used for political communication at the time and TikTok was launched a year later in 2016. Over 80% of the population in Serbia uses the internet to access social media platforms such as Facebook and Twitter (Kovacevic et al. 2023). This applies to most citizens under the age of 65. Gender, income, and educational differences are negligible in these terms (ibid.). There is no available data on the ideological positioning of users of different social media platforms in Serbia.

Six focus groups were conducted in September 2020 in Belgrade, Serbia to collect data from face-to-face interactions regarding the #sedamhiljada initiative and the legacy of the Yugoslav wars of the 1990s. Data from social media interactions (Facebook and Twitter) was obtained using keywords, including #sedamhiljada and Srebrenica, for the period between April 2015, when the initiative emerged, and August 2015, a month after the commemoration. Polls show that attitudes in Serbia towards Srebrenica had not shifted between 2015 and 2020 (Ipsos Public Affairs 2011, 85; Mihajlovic and Lazarevic 2017, 41), which makes focus group and social media data comparable. Focus groups involved 4–6 participants, a total of 39 individuals all of whom lived in various cities and towns in Serbia, recruited through the snowball technique (Goodman 1961) – based on personal networks. Even number of men and women was recruited, aged between 20 and 70, from diverse backgrounds and ideological positionings on the left- and right-wing spectrum. This ensured the representation of diverse voices and perspectives like those that exist online, even though participants were not directly asked for their views on the #sedamhiljada initiative before being selected to participate in focus groups in order not to be primed. Participants were told the topic of the discussion would be the legacy of the Yugoslav wars and peacebuilding. I was the focus group moderator and therefore refrained from sharing my thoughts, evaluating other people’s opinions, and asking leading questions. I was arguably perceived as a cultural insider, which may have enabled more open interactions, where participants felt more at ease to share their intimate thoughts on the topic. The visible presence of the moderator may have further facilitated a more civil discussion of contentious topics. Out of 39 participants, 9 were human rights activists involved in the #sedamhiljada initiative or other similar initiatives dedicated to peacebuilding and reconciliation following a violent conflict. All data was recorded, transcribed, and translated from Serbian to English. Over 600 Facebook and Twitter posts were collected. The ethnicity of the people who participated in discussions on social media that I analysed was determined based on their given and family names as well as their self-identification by using words such as ‘us’ and ‘ours.’

Interactions were a unit of analysis, which means that the focus was on information that unfolded during discussion and on how perspectives were engendered in this process, rather than findings as the result of conversations (Cyr 2016, 235). This also means that this study did not count the number of people who engaged in the interactions on social media. The aim was to explore what interactions revealed about dominant discourses about the legacy of war and how discourses shifted. Focus groups are an appropriate method for studying group dynamics and interactions (Cyr 2016). Critical discourse analysis was then applied to interpret the textual data of focus groups and social media interactions. Discourse analysis studies meanings of text, talk, and social practices more generally (Gill 1996; Van Dijk 1993). It understands language as constructive of reality, rather than reflecting an underlying reality (Gill 1996, 141, 145). I particularly considered discourses of denial and victimhood (see Cohen 2001; Van Dijk 1992) and what underpinned them. The discourse of denial includes outright denial and all other tactics of downplaying the severity, intent, and character of war crimes (Cohen 2001). Victimhood is part of denialist discourse; it refers to the claim that the counterpart started first, and an offense was committed in self-defense or as revenge (Van Dijk 1992). This study did not quantify the occurrences of denialist discourse as opposed to the acknowledgement, this question remains beyond the scope of this paper (for some insights on this question see Vico 2022). This study has focused on the avoidance of acknowledgement motivated by a stereotype threat as a dominant pattern in both social media and focus group data. Three prominent manifestations of stereotype threats have been derived inductively, i.e. identified as occurring patterns in data. The next section elaborates on each of them.

## Findings and analysis

I have identified three prominent manifestations of a stereotype threat observed in both social media and in face-to-face interactions that can be categorised as: ‘genocidal people,’ ‘black and white world,’ and ‘we are being looked at.’ These manifestations signal stages through which a stereotype threat, i.e. the perception of being stereotyped, is constructed: it presupposes that all elements of one group inevitably have characteristics of the group (see Hogg and Abrams 2003), a duality of good and evil, and an audience of outsiders. Most participants perceived that the Serbs had been stigmatised based on the war crimes committed by the members of their ethnic group. Many stated that, consequently, any recognition of the genocide in Srebrenica would mean the affirmation that Serbs were ‘genocidal people.’ They also talked about a lack of recognition of the Serbian victims in the Yugoslav wars of the 1990s by the international society and how one was classified according to their national identity as either good or bad, creating a ‘black and white world.’ Finally, most participants said that their nation was being scrutinised by the international society and that this shaped what was permitted to be said publicly or among cultural outsiders.

While most participants, both those who supported and opposed the initiative, shared perceptions that the Serbs had been stereotyped negatively by the international community because of Serbs’ conduct in the Yugoslav wars of the 1990s, this perception had different effects on the acknowledgement of ingroup responsibility for war crimes on social media compared to face-to-face encounters. Participants in focus groups showed a greater degree of self-criticism and self-reflection in terms of Serb’s conduct in the war and were more willing to acknowledge wrongdoing than those on social media. Those who supported the initiative on social media felt more strongly compelled to defend their position to make a claim they were not ‘bad ambassadors,’ addressed to domestic audiences. Overall, as interactions revealed, people were less willing to be critical publicly because of the exposure to audiences of cultural outsiders, fearing the acknowledgement may legitimise the negative stereotypes of their group in the eyes of outsiders rather than pave the path to reconciliation, and, to some extent, because of the fear of being judged by domestic audiences as ‘bad ambassadors.’

### Perceptions of being labelled as ‘genocidal’ people

The fear of being stereotyped was expressed most strongly in discussions on social media and in face-to-face encounters about whether guilt for war crimes is collective or individual. It can be found in comments such as ‘these crimes were not committed in *our* name,’ and more strikingly in indications that any acknowledgement that the crimes in Srebrenica constitute genocide would imply that Serbs are ‘genocidal people,’ as the following interaction on Facebook shows.

Saša: I want to ask my friends who posted [on Facebook] a slogan ‘Don’t forget Srebrenica’. As a member of ‘genocidal people’, what does it mean? Does it mean that Serbs are ‘shit’?

Marina: How do you in Belgrade know that this [Srebrenica] is fabricated? Come to see an empty town, empty houses … Srebrenica is, unfortunately, true.

Saša: Is it true that Serbs are genocidal?

Marina: Nooo! Have a look at the declaration by the EU Parliament, there it says who is responsible. Not all Serbs, for God’s sake.

By posing the question ‘as a member of genocidal people’ in relation to the banners for remembering Srebrenica on Facebook, Saša shares the perception that Serbs are stereotyped as genocidal by the international society and that initiatives such as #sedamhiljada contribute to the collectivisation of guilt and a negative stereotyping of the entire ethnic group due to the group’s war conduct. When confronted by Marina who confirms that Srebrenica is true, he again directly asks if it is true that Serbs are a genocidal nation. Saša thereby implies that the recognition of the genocide reinforces the negative stereotype about the Serbs. People who supported the #sedamhiljada initiative on social media also showed awareness of the negative stereotype, as the following example shows.

Marko: Individuals committed crimes. They should be held responsible. I am sorry for the victims and wish these crimes never happen again. Before anyone raises the question of the label of genocidal people, I can say that only individuals can be labelled as such, they cannot be defined based on their race, religion, or nationality. Only chauvinists and racists will label broad social groups, such as ethnic or religious, as genocidal because war criminals are part of them.

(Facebook)

In his Facebook post, Marko felt compelled to justify his support for the initiative by de-collectivising guilt and debunking the label of ‘genocidal people’ when he says, ‘individuals committed crimes,’ followed by ‘only chauvinists and racists will label broad social groups … as genocidal … ’ By this, he tries to dismantle the perception that the acknowledgement of crimes in Srebrenica affirms a negative stereotype and to preempt the accusations of his ingroup that he poses a stereotype threat by supporting the initiative (Cohen and Garcia 2005). What can be observed in social media interactions is either outright denial or a strong need to justify one’s support of the initiative. In both cases, there is a closure of debate, leaving little or no room for negotiation. This can be described as ‘exhibitions’ of self-representation (Hogan 2010), a ‘product’ of asynchronous communication on social media, as opposed to performances, a characteristic of synchronous face-to-face communication. Interactions on social media are more strategic and more staged (Marwick 2012), consequently, there is less willingness to revise one’s views. This quote further shows that domestic audiences may also play a role in one’s willingness to acknowledge ingroup responsibility for war crimes, alongside foreign audiences. Focus group participants expressed the same concerns about collective guilt, but were more open to negotiating meanings and did so in a more self-reflective and self-critical manner, as the following example illustrates.

Natalia: It always hurts me, I do not know why when somebody says it was genocide in Srebrenica. Then I always weigh, ‘What was then the suffering of Serbs in Jasenovac.’^2^ I always need to level it up.

… 

Here [referring to the #sedamhiljada initiative], we come to the question of responsibility and whether it can be collective or individual, whether individuals should be tried or whether the people should be declared this way or that way.

(Focus group)

Although Natalia does not recognise the genocide in Srebrenica, she is self-reflective about her stance when she admits that she always needs to equalise war crimes committed by the Serbs and other ethnic groups. When referring to the #sedamhiljada initiative, she poses a question in neutral terms if the initiative promotes collective responsibility and if it justifies negative stereotypes about the Serbs, implying the stereotypes of ‘genocidal people.’ By this, she opens the debate rather than closes it with a strongly opinionated statement. Petar’s account provides similar insights.

Petar: There is no collective responsibility. Individuals are tried. I feel sorry for the Serbian and Croatian people. If Croats had been convicted, the Serbian society would have developed much better, we would have come to terms with what happened together – that there was this and that. But now, it is impossible because they [the Croats] think it [war crimes committed against the Serbs] was justified.

Despite his opposition to the #sedamhiljada initiative, he also expresses regret and partial acknowledgement by implying the Serbs committed war crimes when he says ‘if the Croats had been convicted, … we would have come to terms with what happened together.’ This further shows how denial is motivated by the group reputation threat (Shapiro 2011), i.e. the perceptions of collective guilt. These insights contradict Kasadha’s (2020) arguments about the superior role of social media in promoting post-conflict justice and reconciliation. While social media may provide spaces for people to come together to discuss the legacies of war, as Kasadha (2020) argues, the role social media in facilitating shifts in perceptions about war crimes and promoting acknowledgement proves to be limited compared to face-to-face interactions. Studies of online interethnic contact also argue that online contact is only useful when face-to-face contact is constrained or completely absent (Zezelj et al. 2017).

### Black and white world

The stereotype threat was also frequently manifested in the perception of a ‘black and white world.’ It is the perception the international society, primarily Western countries, holds only Serbs accountable for war crimes of the 1990s and other ethnic groups from the former Yugoslavia as victims, thereby creating a duality of good and evil along ethnic lines. This is observed in Petar’s statement, ‘if Croats had been convicted for war crimes,’ meaning if the Serb victims had been recognised, the Serbs would be more willing to recognise war crimes committed by ethnic Serbs. In a similar vein, Igor replies in annoyance in a comment section below a Facebook post on the official #sedamhiljada Facebook page, ‘The problem for me is that the Serbs are always depicted as aggressors and Muslims as innocent victims.’ Nemanja's condemnation of the initiative reveals the same motivation:

Nemanja: Wars were fought, and people were killed on all sides. When the war ended, everyone fell silent. Only Boris Tadic [former Serbia’s president] went and talked about responsibility and apologised. (…) only Serbs apologised, and only Serbs were convicted (…) No one else, but Serbia. So, to do the right thing does not mean to do the right thing for your country and your people, because other peoples are not much better, but they behave like they are and that is how they are treated eventually.

Nemanja first draws on the denialist discourse’s redistribution of responsibility (Cohen 2001) when he says ‘wars were fought, and people were killed on all sides’ meaning everyone committed war crimes, hence no one is responsible. He then partially acknowledges war crimes committed by the Serbs when he says ‘to do the right thing’ referring to apology and acknowledgement of responsibility. But then he says it should not be done because it legitimises the image of a ‘black and white’ world, where Serbs are depicted as perpetrators and other ethnic groups as victims. His statement is addressed to other Serbs as a criticism of their support of the initiative, expressing a fear that other members of the group could reinforce this negative stereotype about the group. This is an example of a collective threat (Cohen and Garcia 2005), a type of stereotype threat that presupposes someone else’s action, rather than one’s own, can trigger a stereotype about the group. Focus group participants shared the same perceptions:

Boris: A binary system has been created, you are either a good or bad guy, and everyone is classified on a national basis as part of a team that is good or bad.

Sofia: It concerns the prevailing discourse in the West that I have experienced many times from highly educated people who are not experts on this topic and get informed from the mainstream media in the West. They have a black-and-white picture of events in the former Yugoslavia, where Serbs are criminals, Croats, and Muslims are victims, with very little room for nuance.

Boris: That is right.

Sofia: Then, even someone who is not inclined to nationalism has some natural defence mechanism when confronted with that black-and-white picture. Although I am always on the other side of the debate in my country [critical], in the West I caught myself turning into a Serbian nationalist. What should encourage reconciliation is causing a revolt.

[Everyone chuckles affirmatively and replies ‘yes’]

Sofia’s account indicates the origins of these perceptions that Serbs have been negatively stereotyped by the international society. As Sofia says, it is through her personal experiences of interacting with people in the West and Western media that she has formed a perception of a black-and-white world in which Serbs are portrayed exclusively negatively. This can be explained by the fact that, unlike most conflicts that had a dyadic nature, the conflict in the former Yugoslavia was not dyadic (see Kostovicova 2009; Krainin and Ramsay 2022), it was taking place between more than two groups. For instance, in Bosnia, all three ethnic groups were opposed to one another – Serbs, Croats, and Bosniaks.

The fear of own-reputation threat (Shapiro 2011) – that one may be judged in light of negative group stereotypes rather than on individual merits – becomes clear when Boris says, ‘everyone is classified on a national basis.’ This shows the perception that people are often categorised on the basis of national identity and viewed in the light of this identity. Identities are not completely a matter of choice but are shaped in relation to others who try to impose different meanings onto oneself, whereby individual and nation-state levels become permeated. This then leads Sofia to say she has a ‘natural defence mechanism when confronted with that black-and-white picture,’ proving how a stereotype threat hinders the acknowledgement of war crimes even among people who may otherwise be critical of their country’s conduct. Everyone chuckled affirmatively, showing widespread agreement on how stereotypical images of one’s nation could, even if unwittingly, lead to the reproduction of a dominant discourse of denial.

The difference between the Facebook post and this face-to-face interaction is that most people in focus groups maintained a greater degree of self-criticism and self-reflection, observed when Sofia says, ‘In the West I caught myself turning into a Serbian nationalist’ or when she recognises it is a ‘defence mechanism’. There is an undertone of detachment in Boris’s comment in a face-to-face interaction. His comment is more descriptive than prescriptive, unlike Nemanja’s Facebook post, which leaves more room for discussion and negotiation of meanings. This example of face-to-face interaction also shows recollections of personal experience and collective meaning-making, a sense of intimacy and openness about otherwise compromising topics, which was absent in highly visible interactions on social media.

### ‘We are being looked at’: under the moral watch of the international community

This rift between face-to-face and social media interactions regarding the war legacy and the #sedamhiljada initiative can be explained by the concept of cultural intimacy (Herzfeld 1996). Sofia’s comments illustrate how criticism of one’s country is reserved only for cultural insiders. This is observed when Sofia says, ‘in my country’ as she feels compelled to defend her country’s reputation ‘in the West’. This reveals how stereotypes pose a threat to one’s social status and reputation by posing a threat to their country’s reputation. It is not just the preservation of self-esteem that motivates people in this context, as other scholars have pointed out (Cehajic-Clancy et al. 2011; Chung and Woo 2015). Furthermore, this shows the little-understood ways in which state and individual levels become interconnected and interwoven with the promise of international visibility, such as on social media.

‘The West’ here embodies international society, a moral arbitrator in international relations, consisting of the leading (not necessarily English-speaking) countries of the EU and North America. ‘The West’ is seen as the epitome of progress and distinction that less developed nations strive to become part of and seek approval from (see Hatuel-Radoshitzky and Jamal 2022; Herzfeld 1996), which validates its role of a ‘moral arbitrator’. In an era of the proliferation of social media supported by smartphones and 4G and 5G internet connections, one does not need to travel to be exposed to international audiences, this exposure can be found online. Even though most tweets and Facebook posts about the #sedamhiljada initiative and Srebrenica were in Serbian, the translation button integrated into most posts on Facebook and Twitter makes content easily comprehensible to foreign audiences. The ability to retrieve and spread this content using hashtags and share buttons makes the content visible and accessible beyond one’s friends’ circles and networks for public profiles or posts.

The acute awareness of being watched on social media was also observed in Nemanja’s Facebook comment to the #sedamhiljada post. He begins with the remark ‘because it is public’, implying there is a distinction between what is said privately and publicly. Most people post for abstract audiences, family and friends (Litt and Hargittai 2016, 6), but they are also aware that on social media different audiences and contexts may collapse because traces of past activities stick around, and it is harder to keep them separate compared to face-to-face encounters (Marwick and boyd 2010). Research also suggests that the inability to always customise one’s audiences can lead to not sharing (Litt and Hargittai 2016), which may explain why many focus group participants, who knew about the #sedamhiljada initiative, were not posting about it on social media. Visibility to a wider audience was arguably unwanted due to a fear of collective threat (Cohen and Garcia 2005) in the instance of domestic audiences and/ or a group-reputation threat (Inzlicht and Schmader 2011) in the instance of international audiences.

In his closing remarks, Nemanja adds ‘Other peoples were not much better, but they behave like that [they are] and that is how they are treated eventually,’ implying the international community does not hold them accountable because they choose to cover up rather than acknowledge their wrongdoing. Nation-states commonly seek to distance themselves from the legacy of recent civil war and to rebrand their nations as similar to Western European nations (see Rivera 2008), which complements peripheral nations’ broader concern with their image internationally (see Volcic and Andrejevic 2011). Rivera (2008) shows that Croatia removed the legacy of the war from the representations of the country’s history in tourist brochures and cultural policies, and instead positioned itself as exclusively European, while Croatian people intimately did not necessarily identify this way. Similarly, Kosovo’s government has launched several campaigns, including Saatchi and Saatchi's $7.3 billion-worth campaign and #InstaKosova, to rebrand its identity as a young nation with an emphasis on cuisine, natural resources, and architecture, and thereby disassociate itself with the legacy of war (Brantmeier et al. 2020). I find that this translates into how individuals publicly relate to the war legacy to manage their reputation at an international level, demonstrating the interconnectedness between the individual, state, and international levels.

The perceptions of being judged by the international society also came up in focus group interactions. Petar disapproved of the #sedamhiljada initiative by saying ‘it leads to that a foreigner comes and tells me “I have heard of you, war criminals.”’ Similarly, Stefan wrote on Facebook ‘that civil society should not snitch on Serbia to other countries so that other countries can put pressure on Serbia when it suits their interests.’ These comments express concern about exposing ‘dirty laundry before a foreign audience,’ a key aspect of the concept of cultural intimacy, signalling ‘a deep sense of cultural and political vulnerability’ (Herzfeld 1996, 95). They show how the affordance of visibility can have unintended negative effects on the acknowledgement of war crimes, whereby greater visibility can mean a lesser willingness to make such an acknowledgement.

National identities are often constructed in opposition to something else, often an external enemy (e.g. Billig 1995; Guibernau 2004), which could mean the negative stereotypes are paradoxically used domestically to strengthen a sense of national identity and national unity. Nevertheless, the fact that the perception of stereotype threat is shared among most research participants, including those who supported the initiative but who felt urged to distance themselves from such stereotypes, suggests that it is a relevant subject of inquiry and that it poses an obstacle to acknowledgement. These findings make two important theoretical contributions: they show the deep interconnectedness between individual, national, and international levels, and the contextuality of the social media affordance of visibility. This builds on my previous work on national identity among diaspora (Vico 2020a) and supplements the literature that has pointed to the negative consequences of visibility (Mihelj et al. 2023) and relational nature of social media affordances (Willems 2021). The interconnectedness argument means that individuals may identify with their nation not only when that identity is central to them, but also when they realise their reputation and social standing may be dependent on that of their nation, i.e. that their destinies are interwoven or interdependent. The contextuality of visibility argument proposes that implications of social media affordances should be understood at the intersection of platforms’ technical properties such as share button and hashtag, socio-political contexts in which they are embedded such as contentiousness of post-conflict societies, and users’ agency – individual’s understanding and perceptions of platforms and purposes. The socio-political contexts play a strong role in shaping practices and in understanding the social and political consequences of social media.

## Conclusion

Online memory activism has increasingly become the subject of scholarly investigation. It has been mainly hailed for its positive role in disseminating alternative views of the legacy of war (Fridman and Ristic 2020), otherwise constrained in the public sphere. Studies have also pointed to its negative consequences, such as a lack of substantive dialogue (Ellis and Maoz 2007). However, his literature has overlooked the consequences of heightened visibility that social media afford on people’s willingness to acknowledge human rights violations committed by an in-group. This acknowledgement is often deemed key to reconciliation between confronted groups (Cehajic and Brown 2010). Yet, studies show that most nations opt to conceal their war legacy, because such events are deemed reputationally damaging for those countries and accordingly may have material implications (Rivera 2008). In this paper, I have explored the little-understood unintended symbolic implications of mediated visibility on social media and relatedly the role of social media in facilitating acknowledgement.

Drawing on discourse analyses of face-to-face (collected through focus groups) and social media (collected by using keywords) interactions in Serbia regarding the #sedamhiljada initiative, I have found that the fear of being stereotyped negatively, called a stereotype threat (Inzlicht and Schmader 2011), hindered acknowledgement, especially in interactions on social media. Applying Herzfeld’s (1996) concept of cultural intimacy, I have explained that a stereotype threat is particularly threatening on social media because of the exposure to foreign audiences as moral arbitrators. Consequently, people were less willing to acknowledge war crimes committed by an ingroup on social media compared to face-to-face interactions.

This work advances existing understandings of social media practices and consequences in the context of post-conflict justice and reconciliation, and the motivations that impede acknowledgement among so-called ordinary individuals (as opposed to state actors). This study has shown there is a profound interconnectedness between individuals and nation-states, regardless of the strength of an individual’s national identity. Individuals may unwittingly share the destiny of their country when they perceive that they may be viewed in light of their nation’s image. As a result, they may feel compelled to defend their nation’s image publicly – on social media – even if intimately they may be critical of their nation’s conduct. Thereby, this work has underlined the symbolic implications of stereotype threat, such as those to social standing and reputation. The paper has found that social media have a limited role in promoting a discourse shift about the legacy of war due to greater visibility and has thereby highlighted the unintended negative consequences of visibility on social media in post-conflict contexts. The findings supplement existing studies (e.g. Willems 2021) that challenge the presumption of universalistic social media affordances, pointing at their relational or context-dependent characteristics.

Other responses to the identified stereotype threat (besides avoidance of acknowledgement) could also be possible, such as a compulsion to hide one’s national identity or to identify differently. I have observed this tendency in my previous work on diasporas’ communication practices and identity performances (see Vico 2020a). However, this tendency did not emerge in the present study. Stereotype threat (and more broadly, identity threat) can also arise from the fear of being accused of being a ‘bad ambassador’ for one’s group. Visibility to international audiences on social media is not the only explanation for the avoidance of acknowledgement on social media as opposed to face-to-face interactions. People may be discouraged from discussing contentious topics on social media because of the hostile environments and lack of dialogical communication (see Vico 2022). These insights should not replace but complement existing knowledge of the role of social media in facilitating the acknowledgement of ingroup responsibility for war crimes. It contributes to a better understanding of the myriad of motives why people may not participate online or may be reluctant to move toward the acknowledgement of ingroup responsibility.

This research has taken an approach to interactions as a unit of analysis, focusing on ideas and perspectives that emerged during interactions. This meant that participants were not asked to discuss their social media practices in focus groups specifically, but that their interactions on social media were compared to those in focus groups. Hence, future work could consider participants’ perspectives on their social media uses. Future work could also investigate the relationship between domestic media portrayal of the events discussed in this paper and public perceptions of being stereotyped to understand if and to what extent media’s shaming strategies directed to those who publicly contemn war crimes committed by an ingroup shape public understanding of what is permitted to be said publicly. This study has implications for civil society and human rights activists in terms of better understanding the opportunities and challenges of using social media to promote post-conflict justice and reconciliation. Social media are an important means of reaching out to people who may not necessarily be interested in politics, as well as important arenas for public debate when other spaces are constrained and limited.

## Notes

1 The paper has undergone an ethics review process and gained approval from the Research Ethics Committee (ref. no. 630b). Informed consent has been obtained from the participants. All data has been anonymised and quoted under pseudonyms.

2 Jasenovac was a Nazi concentration camp established by the Independent State of Croatia during the Second World War where hundreds of thousands of Jews, Serbs, Roma, and Bosnian Muslims and Croatian opponents of the regime were killed.
